# Detection and pathological value of papillomavirus DNA and p16^INK4A^ and p53 protein expression in cervical intraepithelial neoplasia

**DOI:** 10.3892/ol.2014.1791

**Published:** 2014-01-14

**Authors:** JINGBO WU, XIAO-JING LI, WEI ZHU, XIU-PING LIU

**Affiliations:** 1 Department of Pathology, The Fifth People’s Hospital of Shanghai, Fudan University, Shanghai 200240, P.R. China; 2 Department of Pathology, School of Basic Medical Sciences, Fudan University, Shanghai 200032, P.R. China

**Keywords:** immuhistochemistry, human papillomavirus, p16^INK4A^, p53, cervical intraepithelia neoplasia

## Abstract

The aim of the present study was to investigate human papillomavirus (HPV) infection and p16^INK4A^ and p53 protein expression, to evaluate their roles in the pathological diagnosis and grading for cervical intraepithelial neoplasia (CIN). The detection of HPV DNA and p16^INK4A^ and p53 protein expression were examined in a panel of clinical tissue samples using polymerase chain reaction or immunohistochemistry. In 104 cases, HPV16/18 DNA was identified in 49.0% and HPV6/11 DNA in 9.6% of cases. While in 203 cases, 74.4% positively expressed p16^INK4A^ and 47.3% positively expressed p53. The expression of p16^INK4A^ exhibited a significantly higher rate in the CIN I group than in the squamous metaplasia coupled with hyperplasia group (SMH; P<0.0001) and the CIN II–III group (P=0.005). A marked correlation was revealed between the band-like staining pattern of p16^INK4A^ and HPV16/18 infection. On the contrary, p53 expression was not found to significantly correlate with CIN grade or the HPV16/18 infection status. These results suggested that p16^INK4A^ expression correlates with a higher grade of CIN and may be used as a diagnostic marker to distinguish between CIN I and SMH, as well as between CIN I and CIN II–III.

## Introduction

Cervical cancer is the second most frequent cause of cancer-related mortality in females worldwide and the most common type of cancer among females in the majority of developing countries (1–3). Currently, clinical management of preinvasive cervical cancer largely relies on histological examination to confirm cervical intraepithelial neoplasia (CIN) and its grading. Generally, higher grade (CIN II–III) cases require active treatment, including cervial conization or cervical loop electrosurgical excision procedure. Although the histological features of CIN are well understood, inconsistent use and misinterpretation of such features may occur due to intra- and interobserver variability (4,5). On the basis of morphology alone, it can be difficult to distinguish between squamous metaplasia coupled with hyperplasia (SMH) and CIN I, and between CIN I and CIN II–III. Moreover, small biopsy size, tangential sectioning, thermal artifact, coexistent inflammatory or reactive lesions and application of subjective criteria all increase the difficulty for the diagnosis of CIN (6). Therefore, objective diagnostic methods in addition to histology are required to accurately diagnose CIN in histological specimens.

Human papillomavirus (HPV) plays an etiologic role in cervical carcinogenesis and is detectable in preinvasive and invasive cervical epithelial neoplasms (7,8). The most common high-risk HPV subtypes include types 16 and 18, which account for ~70% of HPV species detected in cervical cancer. Low-risk types, 6 and 11, account for ~90% of HPV species present in genital warts (1). Infections with low-risk types have been known to cause genital warts and low-grade cervical abnormalities.

p16 is a cyclin-dependent kinase inhibitor that regulates the transition from G1 to S phase of the cell cycle and normally functions as a tumor suppressor (9). Although p16 levels are reduced in a variety of malignant tumors, this gene product has been shown to be upregulated (or overexpressed) in the majority of high-grade cervical dysplasias and carcinomas induced by high-risk HPV subtypes (10). p53, as a tumor suppressor, is one of the major factors controlling cell proliferation. As the ‘guardian of the genome’, it arrests the cell cycle in response to DNA damage or directs the damaged cell to an apoptotic pathway.

To investigate HPV infection and the protein expression of p16^INK4A^ and p53, and to evaluate their potential roles in the pathological diagnosis and grading of cervical CIN, HPV DNA and p16^INK4A^ and p53 expression were examined in a panel of clinical tissues samples (including 40 SMH, 24 cervical condyloma, 120 CIN and 19 cervical cancer) using polymerase chain reaction (PCR) or immunohistochemistry (IHC). In addition, the correlation between p16^INK4A^ and p53 and the degree of CIN with HPV were analyzed.

## Materials and methods

### Tissue samples

Samples were obtained from formalin-fixed, paraffin-embedded blocks of cervical biopsies from 203 patients accessioned at the Department of Pathology, The Fifth People’s Hospital of Shanghai, Fudan University (Shanghai, China), between 2006 and 2012. The age of patients ranged between 12 and 77 years, with a median age of 48 years. None of the patients had been treated for cervical abnormalities prior to the biopsy.

**Figure 1:**
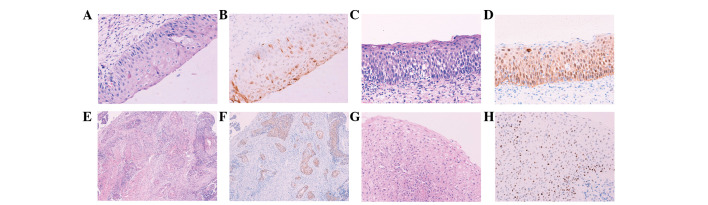
H&E and immunohistochemical staining of various tissue sections. Images were captured under a light microscope at ×200 magnification with the exception of panels E and F (magnification, ×100). Tissue sections presented are (A and B) CIN I, (C and D) CIN II, (E and F) cervical cancer and (G and H) cervical condyloma. (A, C, E and G) H&E staining showing morphologies of all tissues and (B, D, F and H) immunohistochemical staining, showing differential expression of p16^INK4A^ protein. (B) Spotty and (D and F) band-like patterns and (H) strong expression of p53 protein in the cervical condyloma. H&E, hematoxylin and eosin; CIN, cervical intraepithelial neoplasia.

The specimens included 40 cases of SMH, 24 cases of cervical condyloma, 120 cases of CIN (CIN I, 37 cases; CIN II–III, 83 cases) and 19 cases of cervical cancer. All slides were reviewed by at least three pathologists, discrepancies were resolved by reevaluation and discussion, and concurrence was achieved. Only one final diagnosis was determined for each case. Representative images of hematoxylin and eosin (H&E) staining from tissues with increasing grades of cervical lesions are shown in Fig. 1 [images were viewed under a light microscope (BX45, Olympus, Tokyo, Japan)]. The study was approved by the ethics committee of the Fifth People’s Hospital of Shanghai, Fudan University (Shanghai, China). Written informed consent was obtained from the patient’s families.

### DNA extraction

Only 104 specimens of DNA were detected. In total, 10 formalin-fixed, paraffin-embedded tissues were sectioned (5-μm-thick). The tissue sections were first deparaffinized in xylene and grading alcohol, and then digested by proteinase K at 1.25 mg/ml in 400 μl lysis buffer [20 mM Tris (pH 8.0), 10 mM EDTA (pH 8.0) and 2% SDS (pH 7.2)] at 56ºC overnight. The proteinase K was heat inactivated at 95ºC for 10 min, one-third of the volume of saturated sodium acetate (pH 5.2) was added and samples were centrifuged for 15 min at 18,000 × g. Supernatant was transferred into a fresh tube, two volumes of ice-cold 100% ethanol were added and the sample was centrifuged for 15 min at 18,000 × g. Next, the supernatant was discarded and the pellet was washed twice with 75% ethanol. Following air drying at room temperature for 15 min, the DNA pellet was dissolved in distilled deionized water and the DNA solution was stored at −20ºC.

To ensure integrity of the DNA, a 5-μl DNA aliquot was amplified for the β-globin housekeeping gene by PCR with Taq polymerase (Sangon Biotech Shanghai Co., Ltd., Shanghai, China). Primers specific for the β-globin gene were as follows: PC04, 5′-CAA GAG CCA AGG ACA GGT AC-3′; and GH20, 5′-CAA CTT CAT CCA CGT TCA CC-3′. PCR was performed in a 50-μl reaction with 40 cycles of amplification. Each cycle included a denaturation step at 94ºC for 30 sec, primer annealing step at 53ºC for 30 sec and chain elongation step at 72ºC for 40 sec, followed by the final elongation step at 72ºC for 5 min. DNA products were visualized in 2.0% agarose gel.

### HPV detection and typing

The current study focused on the detection of the most common HPV subtypes; high-risk HPV16/18 and low-risk HPV6/11. In total, three various primer sets were used for the PCR detection. For PCR of the HPV6/11 DNA to amplify a 334-bp HPV L1 gene fragment, the following primers were used: 5′-TGC AAG AAT GCA CTG ACC AC-3′ and 5′-TGC ATG TTG TCC AGC AGT GT-3′ (11). PCR reactions were performed in 50-μl volumes containing 5 μl DNA and 2.5 U Taq DNA polymerase using the following conditions: Initial 5-min denaturation step at 94ºC, followed by 40 cycles of amplification in a PCR processor (PTC-100; MJ Research Inc., St. Bruno, QC, Canada). Each cycle included a denaturation step at 94ºC for 30 sec, primer annealing step at 51ºC for 30 sec and chain elongation step at 72ºC for 40 sec, followed by a final elongation step at 72ºC for 5 min. For the detection of HPV16 and HPV18, the following specific primers were used: HPV16, 5′-TGA GCA ATT AAA TGA CAG CTC AGA-3′ and 5′-GA GAA CAG ATG GGG CAC ACA AT-3′; and HPV18, 5′-GAC CTT CTA TGT CAC GAG CAA TTA-3′ and 5′-GC ACA CCA CGG ACA CAC AAA G-3′ (12). The HPV16 primers amplified a 212-bp fragment and the HPV18 primers amplified a 236-bp fragment. HPV16/18 PCR reactions were performed under conditions identical to PCR for HPV6/11, with the exception of the annealing temperature of 54ºC. All PCR products were subjected to electrophoresis in a 2% agarose gel at 100 V for 30 min. The 334- or 212/236-bp product was visualized with ethidium bromide staining. Additional confirmation of the amplified HPV-specific sequence was performed by DNA sequencing (Shanghai GeneCore BioTechnologies Co., Ltd., Shanghai, China). Of note, the absence of HPV6/11 or HPV16/18 PCR products did not exclude the presence of other HPV subtypes, which was not assessed in the present study.

### Immunohistochemical staining

For the detection of p16^INK4A^ and p53 protein expression, IHC was performed using the Chemmate™ EnVision™ detection kit (DakoCytomation, Glostrup, Denmark). The sections were dewaxed in xylene, rehydrated in a series of gradient alcohol solutions and rinsed in water. Sections were then treated by two immersions in a 3% hydrogen peroxide bath in absolute methanol (5 min each) to inhibit endogenous peroxidase activity, followed by rinsing in water. Antigen retrieval was accomplished by heating the specimen in 0.01 M citrate buffer (pH 6.0) in a high-intensity microwave oven for 25 min. Monoclonal antibodies against p16 (clone 16P07; 1:100) and p53 (clone DO-7; 1:100) (Shanghai Changdao Biotech Co., Ltd., Shanghai, China) were applied for 12 h at 4ºC. The sections were sequentially incubated with secondary antibody at 37ºC for 30 min and carefully rinsed with several changes of TBS between each step. The sections were then incubated with 3,3′-Diminobenzidene (DakoCytomation) and lightly counterstained with Harris hematoxylin for 60 sec. Sections of the p16^INK4A^-positive cervical cancer and p53-positive breast carcinoma were included to serve as the positive controls for p16^INK4A^ and p53 IHC, respectively. Negative control sections were processed by eliminating the use of respective primary antibodies.

### Evaluation of immunohistochemical staining

Nuclear and/or cytoplasmic staining in >10% of atypical cells was interpreted as positive for p16^INK4A^; cytoplasmic staining alone was considered non-specific and interpreted as negative.

Brown staining in the nuclei of >10% of atypical cells was indicated as positive for p53. For cases with condyloma, CIN and/or cervical cancer, p16^INK4A^ and p53 were evaluated in the areas exhibiting the highest grade of atypia or dysplasia. The immunostained slides were reviewed by at least three pathologists and a consensus was achieved. Staining patterns were found to correlate with the respective H&E diagnoses. The two staining patterns observed in p16 staining were band-like and spotty. The staining pattern was recorded as band-like when >90% of contiguous squamous cells stained positive or spotty when >10% of scattered squamous cells stained positive (6).

### Statistical analysis

Statistical analysis was performed using χ^2^, Fisher’s exact or Spearman’s rho tests. SPSS software (version 13.0; SPSS, Inc., Chicago, IL, USA) was used for all statistical analyses. For all tests, P<0.05 was considered to indicate a statistically significant difference.

## Results

### Detection of HPV-specific DNA by PCR

**Figure 2:**
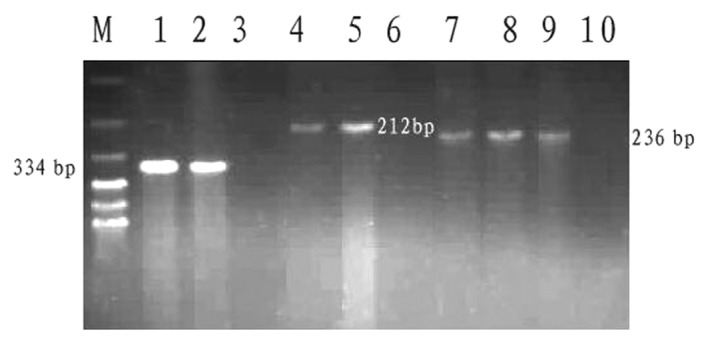
Images of agarose gel with polymerase chain reaction products for various HPV DNA species. DNA bands in the following lanes present an amplified product specific for viral DNA: M, 100-bp DNA ladder (molecular weight marker); 1, HPV6/11 subtype; 2, HPV6/11-positive control; 3, HPV6/11-negative control; 4, HPV16 subtype; 5, HPV16-positive control; 6, HPV16-negative control; 7 and 8, HPV18 subtype; 9, HPV18-positive control; and 10, HPV18-negative control. HPV, human papillomavirus.

**Figure 3:**
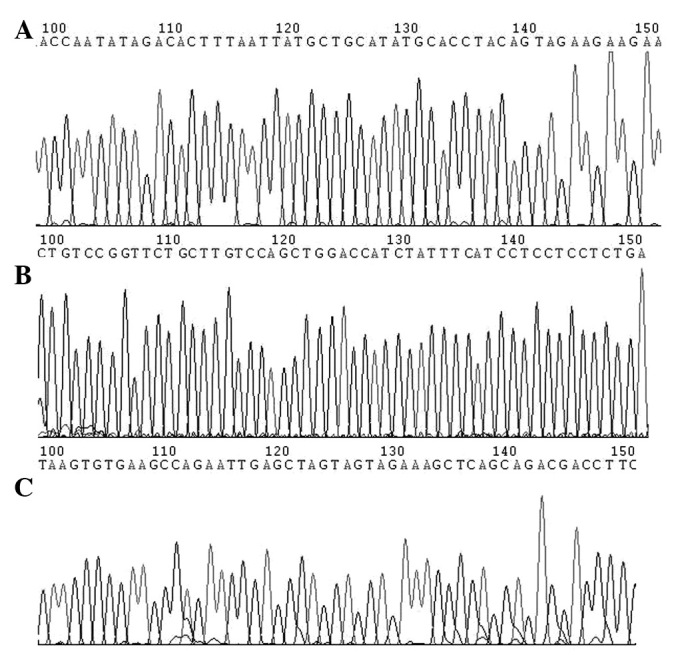
Chromatographic view of sequencing results for HPV-specific DNA products. (A) HPV6/11, (B) HPV16 and (C) HPV18. HPV, human papillomavirus

DNA prepared from previously collected cervical tissue samples were pre-tested for their integrity and PCR performance. The β-globin housekeeping gene DNA was detected in all 104 samples (including 12 SMH, 10 cervical condyloma, 65 CIN and 17 cervical cancer). Representative DNA products amplified with HPV subtype-specific primers are shown in Fig. 2. Each PCR reaction produced only one correct band with the predicted size, indicating the specificity of PCR amplifications. Positive DNA products were confirmed for their correct nucleotide sequences by DNA sequencing (Fig. 3).

Results of all PCR detection are summarized in Table I. DNA sequences for the HPV6/11 subtype was amplified from 10/104 (9.6%) cases and its frequency in the cervical condyloma group was significantly higher than those in other groups (P<0.05). The HPV16/18 subtype was identified in 51/104 (49.0%) cases. Of the 51 HPV16/18-positive cases, 31 were HPV16, 15 were HPV18 and double-positive expression of HPV16/18 was identified in five cases. The HPV16/18 frequency in tissues increased in the following order: SMH, cervical condyloma, CIN I, CIN II–III and cervical cancer. Of those infected by HPV16/18, 48 (94.1%) cases were diagnosed with CIN or cervical cancer.

HPV16/18 detection frequencies were not significantly different between the CIN II–III and CIN I groups, or between the cervical cancer and CIN II–III groups (all P>0.05). However, the HPV16/18 frequency was significantly higher in the CIN I group than that in the SMH group (P=0.018).

### p16^INK4A^ expression by immunohistochemical staining

The p16^INK4A^ expression was located in the nucleus and/or cytoplasm and exhibited two staining patterns, scattered spotty and contiguous band-like expression. The SMH and cervical condyloma groups were negative for p16^INK4A^ expression or exhibited predominately spotty expression. The CIN I group exhibited similar spotty expression to the SMH and condyloma groups, or contiguous band-like expression (Fig. 1A and B). By contrast, the CIN II–III and cervical cancer groups showed mainly contiguous band-like patterns (Fig. 1C–F). p16^INK4A^ expression was not observed in squamous and glandular epithelia adjacent to the dysplastic lesions coexisting in the same tissue sections.

The results of the p16^INK4A^ staining are summarized in Table II. p16^INK4A^-positive expression was observed in 151/203 (74.4%) cases examined. Of the 151 cases, 114 (75.5%) revealed the band-like positive pattern and 37 (24.5%) showed the spotty, positive pattern. The p16^INK4A^ positive expression rates were significantly higher in the CIN I group (81.1%,) than in the SMH group (32.5%) (P<0.0001), and significantly higher in the CIN II–III group (95.8%) than that in CIN I group (P=0.005). However, the p16^INK4A^-positive expression was not significantly different between the cervical cancer and CIN II–III groups (P=0.738). The expression patterns of p16^INK4A^ changed from the scattered spotty to contiguous band-like pattern with increasing grade of cervical lesions. In addition, a significant difference was achieved in the p16^INK4A^ staining pattern (band-like vs. spotty or negative) among cervical tissues with various stages of lesion progression (P<0.0001; Table II).

### p53 expression and its correlation with p16^INK4A^ protein by IHC

The p53 expression was localized in the nuclei and positive expression was identified in 47.3% (96/203) of the cases examined. The strong p53 expression was observed in cases of cervical condyloma with positive HPV6/11 (Fig. 1G–H). However, p53 staining was not observed in the normal epithelium coexisting in the same tissue sections.

The rate of p53 positive expression in cervical condyloma was significantly higher than that in the other groups (P<0.05), with the exception of the cervical cancer group. Results of p53 expression during lesion progression are shown in Table II.

In addition, the correlation between p16^INK4A^ and p53 protein expression were analyzed by the Spearman’s rho test (Table III) and no correlation was identified between the expression of the two proteins (r=0.6669; P=0.2189).

### Correlation between p16^INK4A^ or p53 expression and HPV infection

Table IV shows that p16^INK4A^ expression was found to correlate with HPV infection in every grade of cervical lesion. The p16^INK4A^-positive expression was observed in 47 cases in which HPV16/18 was also found to be positive. Of the 47 lesions, 42 cases (89.4%) showed p16^INK4A^ band-like expression patterns and the remainder (10.6%) showed spotty expression patterns or were negative for p16^INK4A^. The presence of spotty and band-like expression was found to correlate markedly with HPV16/18 infection (r=1.0000; P<0.0001). In addition, the presence of the band-like expression alone was found to correlate with HPV16/18 infection (r=0.9747; P=0.0048). However, no significant correlation was identified between spotty or negative p16^INK4A^ expression and HPV16/18 infection (r=0.2294; P=0.7015). Of the 10 cases that positively expressed HPV6/11, 5 (50.0%) were p16^INK4A^-positive with spotty staining pattern. No correlation was observed between p16 ^INK4A^ staining and HPV6/11 infection (r=−0.5643; P=0.3217).

Widespread expression of p53 was observed in lesions infected with HPV6/11 (Fig. 3G and H) and it was more intense than in those infected with HPV16/18. Of the 51 cases infected by HPV16/18, 23 were p53 positive and of the 10 cases infected by HPV6/11, 8 were p53 positive. The presence of HPV infection (HPV16/18 or HPV6/11) was found to significantly correlate with the immunoreactivity of p53 (P=0.044).

## Discussion

To date, >100 types of HPV have been identified from clinical samples. In total, ~40 different HPV genotypes are considered to be sexually transmitted, which may be regarded as causal agents for cervical cancer and CIN. The majority of previous studies have hypothesized that high-grade CIN (CIN II and III) and cervical cancer markedly correlate with the persistence of high-risk HPV types (13,14).

In the present study, HPV16 and HPV18 subtypes were detected with a frequency of 49.0%, mainly in the CIN and cervical cancer groups. HPV16 was predominant in 60.8% of the HPV16/18-positive tissues. No statistically significant difference in HPV16/18 detection frequencies was observed between the CIN I and CIN II–III groups, or between the CIN II–III and cervical cancer groups. However, the frequency in the CIN I group was significantly higher than that in the SMH group (P=0.018). Therefore, HPV16/18 detection was also useful to distinguish CIN and cervical squamous cell carcinoma from SMH.

HPV6/11 was detected in 90.0% of condyloma cases, but only in 5.6% of the CIN I cases, where 55.6% were positive for HPV16/18. Therefore, the difference in the distribution of HPV subtypes was significant between the cervical condyloma and CIN I groups. Since it is well known that prognoses for high- and low-risk HPVs are significantly different, future studies are required to confirm that cervical condyloma and CIN I belong to low-grade squamous intraepithelial lesions in the Bethesda System (2001).

The p16 gene product normally acts to inhibit progression through the cell cycle by binding to cyclin-dependent kinase 4/6, therefore, preventing the phosphorylation and subsequent inactivation of retinoblastoma protein. A previous study by Sakaguchi *et al* was the first to correlate p16^INK4A^ overexpression with cellular malignant behaviors (15). In addition, a previous study by Sano *et al* was the first to describe the use of p16^INK4A^ as a diagnostic marker in the pathology of CIN (16). The majority of studies have since reported a significant correlation between diffuse or contiguous band-like patterns of p16^INK4A^ staining in CIN II–III and the presence of high-risk HPV types. In addition, studies have reported a correlation between negative to scattered spotty p16^INK4A^ staining patterns in CIN I and the presence of low-risk HPV types (17–19). However, in contrast to the majority of studies, certain studies have shown that a significant number of high-grade CIN and squamous cell carcinomas are negative for p16^INK4A^ (20,21), and another study observed diffused or band-like p16^INK4A^ staining patterns in specific cases of CIN I and SMH (22). Possible explanations for such heterogeneity in p16^INK4A^ staining may include the inherent and unavoidable intra- and inter-observer variability in the morphological categorization of CIN, lack of standardization in the scoring of p16^INK4A^ immunoexpression (nuclear and/or cytoplasmic staining; and positive vs. negative distribution within the epithelium), various antibody clones used for IHC and the existence of various HPV geographical variations (23).

In the present study, p16^INK4A^ expression increased gradually with the progression of lesions, and immunostaining patterns changed from the scattered spotty to contiguous band-like patterns. These results indicated a marked correlation between p16^INK4A^ immunostaining and degree of epithelial atypia. In addition, the expression rate of p16^INK4A^ in the CIN II–III group was found to be significantly higher than that in the CIN I group, suggesting the potential use of p16^INK4A^ to discriminate between CIN II–III and CIN I. On the other hand, the expression rate of p16^INK4A^ was significantly higher (P<0.0001) in CIN I, compared with that in SMH. In addition, the immunostaining patterns for p16^INK4A^ were not only spotty, but also band-like in specific cases, suggesting that p16^INK4A^ may also be a potential marker to distinguish between CIN I and SMH.

Previous studies have shown that high-risk HPV may induce p16^INK4A^ overexpression (16,24). One mechanism of which may be due to the negative feedback loop where p16^INK4A^ acts as a cell cycle inhibitor and may be induced in response to the HPV infection to reduce the retinoblastoma function (such as inactivation by HPV E7). However, Kuo *et al* previously hypothesized that HPV-related mucosal dysplasia in various anatomical locations may lead to various molecular pathways (25). To date, the majority of studies have reported that immunoreactivity for p16^INK4A^ may be used as a surrogate marker of HR-HPV and Kong *et al* hypothesized that p16^INK4A^ IHC was superior for the detection of HR-HPV by *in situ* hybridization (26). In the current study, a band-like pattern of p16 immunostaining was found to markedly correlate with HPV16/18 infection, suggesting that a band-like expression of p16^INK4A^ may be induced by HPV16/18 DNA. It appears that the higher rate of the HPV16/18 expression correlates with stronger intensity of p16^INK4A^ staining.

Mutations in the p53 gene are the most frequent mutations encountered in types of human cancer. The majority of studies have found that the presence of p53 in cancer cells correlates with the consequence of p53 mutation due to failure of the mutated protein to transactivate its own negative regulator MDM2. The p53 oncoprotein expression has been detected in carcinomas from various sites, including breast, colon, bladder and lung. However, the correlation between HPV and p53 IHC staining in cervical lesions has been controversial (27,28). In the present study, no significant correlation was observed between p53 expression and cervical lesions. In addition, no correlation was identified between HPV types and the immunoreactivity of p53. However, the presence of the HPV infection by HPV6/11 or HPV16/18 subtype was found to significantly (P=0.044) correlate with the immunoreactivity of p53, which is consistent with a study by Giannoudis *et al* who hypothesized that p53 was more widely expressed in cervical lesions infected with low-risk HPV than those with intermediate or high-risk HPV types (29).

In conclusion, the management of preinvasive cervical diseases remains largely dependent on the confirmation of CIN. However, the current classification of CIN evaluated by histology exhibits a significant overlap in the morphological criteria between SMH and CIN I, as well as between CIN II and CIN II–III. The results of the current study suggested that p16^INK4A^ immunostaining may be used as an additional marker for a more accurate determination of CIN in the cervical lesions.

## Figures and Tables

**Table I:** Polymerase chain reaction-detected HPV subtype-specific DNA in various tissues.

		Positive cases, n (%)	
		
Histological classification	Total cases, n	HPV 16/18 subtype	HPV 6/11 subtype
SMH	12	1 (8.3)	0 (0)
Condyloma	10	2 (20.0)	9 (90.0)
CIN I	18	10 (55.6)	1 (5.6)
CIN II–III	47	26 (55.3)	0 (0)
Cervical cancer	17	12 (70.6)	0 (0)
Total	104	51 (49.0)	10 (9.6)

HPV, human papillomavirus; SMH, squamous metaplasia coupled with hyperplasia; CIN, cervical intraepithelial neoplasia.

**Table II:** Expression of p16^INK4A^ and p53 with the degree of CIN in 203 cervical lesions.

		Expression of p16^INK4A^			
			
Histological classification	n	Band, n	Spotty, n	Total, n (%)	Expression of p53, n (%)
SMH	40	3	10	13 (32.5)	8 (20.0)
Condyloma	24	2	8	10 (41.7)	18 (75.0)
CIN I	37	15	15	30 (81.1)	18 (48.6)
CIN II–III	83	76	4	80 (95.8)	40 (48.2)
Cervical cancer	19	18	0	18 (94.7)	12 (63.2)
Total	203	114	37	151 (74.4)	96 (47.3)

CIN, cervical intraepithelial neoplasia; SMH, squamous metaplasia coupled with hyperplasia.

**Table III:** Correlation between p16^INK4A^ and p53 expression with no regard to tissue type.

p16^INK4A^ expression	Negative, n	Spotty, n	Band (+), n	Band (++), n	Band (+++), n	Total, n
p53 negative	37	18	12	17	23	107
p53 positive	15	19	12	20	30	96
Total	52	37	24	37	53	203

For cases exhibiting a band-like staining pattern, the location of the band was recorded as confined to the lower third (+), confined to the lower two-thirds (++) or full thickness (+++).

**Table IV:** Expression patterns of p16^INK4A^ in 51 HPV16/18-positive and 10 HPV6/11-positive cases.

HPV subtype	n	SMH		Condyloma		CIN I		CIN II–III		Cervical cancer	
				
		Spotty, n	Band, n	Spotty, n	Band, n	Spotty, n	Band, n	Spotty, n	Band, n	Spotty, n	Band, n
16/18	47	0	0	1	0	4	4	0	26	0	12
6/11	5	0	0	5	0	0	0	0	0	0	0
Total	52	0	0	6	0	4	4	0	26	0	12

HPV, human papillomavirus; SMH, squamous metaplasia coupled with hyperplasia; CIN, cervical intraepithelial neoplasia.

## References

[1] Bauer HM, Ault K (2006). Human papillomavirus: Current prevalence and future Protection. Sex Trans Dis.

[2] Chih HJ, Lee AH, Colville L, Binns CW, Xu D (2013). A review of dietary prevention of human papillomavirus-related infection of the cervix and cervicalintraepithelial neoplasia. Nutr Cancer.

[3] Qian JH, Xie X, Yie DF (2006). Epidemiologic features of human paplillomavirus infection in cervix. Chin J Obstet Gynecol.

[4] Martin CM, O’Leary JJ (2011). Histology of cervical intraepithelial neoplasia and the role of biomarkers. Best Pract Res Clin Obstet Gynaecol.

[5] Dascau V, Furau G, Furau C, Paiusan L (2012). Cervical intraepithelial neoplasia in the ‘dr. Salvator vuia’ clinical obstetrics and gynecology hospital - arad during the 2000–2009 period. Maedica (Buchar).

[6] Walts AE, Lechago LJ, Bose S (2006). P16 and Ki67 immunostaining is a useful adjunct in the assessment of biopsies for HPV-associated anal intraepithelial neoplasia. Am J Surg Pathol.

[7] Monsonego J, Pintos J, Semaille C, Beumont M (2006). Human papillomavirus testing improves the accuracy of colposcopy in detection of cervical intraepithelial neoplasia. Int J Gynecol Cancer.

[8] Massad LS, Einstein MH, Huh WK, Katki HA (2013). 2012 updated consensus guidelines for the management of abnormal cervical cancer screening tests and cancer precursors. J Low Genit Tract Dis.

[9] Tsoumpou I, Arbyn M, Kyrgiou M, Wentzensen N (2009). p16(INK4a) immunostaining in cytological and histological specimens from the uterine cervix: a systematic review and meta-analysis. Cancer Treat Rev.

[10] Sano T, Oyama T, Kashiwabara K, Fukuda T, Nakajima T (1998). Expression status of p16 protein is associated with human papillomavirus oncogenic potential in cervical and genital lesions. Am J Pathol.

[11] Sotlar K, Diemer D, Dethleffs A, Hack Y (2004). Detection and typing of human papillomavirus by E6 nested multiplex PCR. J Clin Microbiol.

[12] Gheit T, Tommasino M (2011). Detection of high-risk mucosal human papillomavirus DNA in human specimens by a novel and sensitive multiplex PCR method combined with DNA microarray. Methods Mol Biol.

[13] Shirendeb U, Hishikawa Y, Moriyama S, Win N (2009). Human papillomavirus infection and its possible correlation with p63 expression in cervical cancer in Japan, Mongolia, and Myanmar. Acta Histochem Cytochem.

[14] Lax S (2011). Histopathology of cervical precursor lesions and cancer. Acta Dermatovenerol Alp Panonica Adriat.

[15] Sakaguchi M, Fujii Y, Hirabayashi H, Yoon HE (1996). Inversely correlated expression of p16 and Rb protein in non-small cell lung cancers: an immunohistochemical study. Int J Cancer.

[16] Sano T, Oyama T, Kashiwabara K, Fukuda T, Nakajima T (1998). Immunohistochemical overexpression of p16 protein associated with intact retinoblastoma protein expression in cervical cancer and cervical intraepithelial neoplasia. Pathol Int.

[17] Koshiol J, Lindsay L, Pimenta JM, Poole C (2008). Persistent human papillomavirus infection and cervical neoplasia: a systematic review and meta-analysis. Am J Epidemiol.

[18] Carozzi F, Gillio-Tos A, Confortini M (2013). Risk of high-grade cervical intraepithelial neoplasia during follow-up in HPV-positive women according to baseline p16-INK4A results: a prospective analysis of a nested substudy of the NTCC randomised controlled trial. Lancet Oncol.

[19] Lesnikova I, Lidang M, Hamilton-Dutoit S, Koch J (2009). p16 as a diagnostic marker of cervical neoplasia: a tissue microarray study of 796 archival specimens. Diagn Pathol.

[20] Lee S, Kim H, Kim H, Kim C, Kim I (2012). The utility of p16INK4a and Ki-67 as a conjunctive tool in uterine cervical lesions. Korean J Pathol.

[21] Lin ZH, Liu MZ, Zhao YW, Wu QY (2006). Study of p16 expression and DNA ploidy in HPV-negative cervical cancers and precursors. Chin J Pathol.

[22] Soma M, Kamaraj S (2010). Detection of human papillomavirus in cervical gradings by immunohistochemistry and typing of HPV 16 and 18 in high-grades by polymerase chain reaction. J Lab Physicians.

[23] Samir R, Asplund A, Tot T, Pekar G, Hellberg D (2011). High-risk HPV infection and CIN grade correlates to the expression of c-myc, CD4+, FHIT, E-cadherin, Ki-67, and p16INK4a. J Low Genit Tract Dis.

[24] Yim EK, Park JS (2007). Biomarkers in cervical cancer. Biomark Insights.

[25] Kuo KT, Chang HC, Hsiao CH, Lin MC (2006). Increased Ki-67 proliferative index and absence of P16INK4 in CIN-HPV related pathogenic pathways different from cervical squamous intraepithelial lesion. Br J Ophthalmol.

[26] Kong CS, Balzer BL, Troxell ML, Patterson BK, Longacre TA (2007). p16INK4A immunohistochemistry is superior to HPV in situ hybridization for the detection of high-risk HPV in atypical squamous metaplasia. Am J Surg Pathol.

[27] Bragança JF, Sarian LO, Pitta DR, Maito AB (2008). Expression of p16(INK4a) and cervical infection with high-risk human papillomavirus are not related to p53 activity in cervical intraepithelial neoplasia. Int J Gynecol Cancer.

[28] Türkçüoğlu I, Tezcan S, Kaygusuz G, Atabekoğlu CS (2007). The role of p53, Bcl-2 and Ki-67 in premalignant cervical lesions and cervical cancer. Eur J Gynaecol Oncol.

[29] Giannoudis, Herrington CS (2000). Differential expression of p53 and p21 in low grade cervical squamous intraepithelial lesions infected with low, intermediate, and high risk human papillomaviruses. Cancer.

